# Correspondence

**Published:** 1883-11

**Authors:** 


### Correspondence.

GENEVA, SWITZERLAND,
Sept. 29th, 1883.

*Editor Ind. Prac.:*

It is somewhat difficult in a quiet place like our city of Geneva, to find much of interest to write about, but it is a pleasant place for over-traveled wanderers to obtain the rest and repose so much needed, at times, during their journeyings. It seems but a short time since you yourself, weary and depressed, came to Geneva for a few days quiet, and yet it was two years ago; one cannot realize the flight of time.

I have not forgotten our quiet dinners at *Juges', en l'ile*, where we sat in the little balcony that juts out over the brawling Rhone, whose waters, so deeply blue, have but just parted from those of Geneva Lake, and where at the same time we discussed our *Truite de rivierè*, our *Chambertin*, and our far-away American friends.

Not long since, having contracted what the natives here call the "Grip," *i.e.*, a very severe form of influenza, I went to Vevey, at the head of our lake, for a few days change of air. While there my friend, Dr. De Trey, a Swiss gentleman, a graduate of one of the

Philadelphia dental schools, and one of the finest operators on the continent, was kind enough to show me his method of performing the operation for "resection of the dental pulp." As I have seen no publication of it, I will briefly state what I saw.

The tooth was a second bicuspid; cavity on the posterior approximal surface, a small opening the size of a pin's head into the pulp chamber. The doctor excavated thoroughly, not however touching any portion of the dentine covering the pulp, which stood up in the center like a little mound. With a sharp, thin bladed instrument prepared by himself for these operations, with one stroke he removed that portion of the dentine, together with the portion of the pulp covered by it. After allowing the blood vessels to empty themselves pretty thoroughly, he applied chloride of sodium for a few moments to draw out the remaining blood, and to contract the vessels, washed well with tepid water, dried the cavity thoroughly, and filled immediately with oxy-chloride of zinc, mixed to the consistency of putty. There was but little pain during or after the operation. The doctor does this almost daily. In every case presented to him, in which he may reasonably expect the pulp to die under a gold filling, he prefers resection, knowing that he leaves a healthy wound, and the probability of a rapid cicatrization.

We have a dental school, established and supported by the State, in our city. It has been in operation now about three years. In my next letter I will give you some account of this institution, so that your readers may compare it with our schools at home. A great many Americans have visited in Switzerland this summer, more than for many years past. I have had the pleasure of seeing quite a number of them, recommended to me by their dentist at home, for which courtesy I desire to thank my professional brethren.

A number of prominent dentists from the States have also been here. I had the pleasure of a call from our friend L. D. Sheppard, of Boston. He is looking better than he did two years ago, and still retains a lively recollection of that meeting at Wiesbaden. Speaking of our society, where can you find one that can boast of such a member as our young friend Dr. Miller, of Berlin. There is an example for the rising young men of our profession to follow ; but let me tell them how he passes the time unoccupied by his professional duties. He rises at five o'clock in the morning, goes to his studies at the chemical laboratory, where he works until nine, when his professional labor begins, which lasts until four, with one hour intermission for lunch. At four o'clock he is off again to his studies until sometimes, midnight, forgetting the "whole world and the rest of mankind."

That is the way such men are made ; they must study, work, investigate. No matter how much talent a young man may possess, if he does not cultivate it, it will bear no fruits. The secret of the success of men like Miller is a simple one. It is work, work, work.

A. A. BLOUNT.

---

667 FIFTH AVENUE,
NEW YORK, Sept. 22, 1883.

*Dear Mr. Editor:*

The INDEPENDENT PRACTITIONER is before me. It has the exquisite flavor of independence about it, and meets with my approval. I like the editorials, the interesting reports of society doings in New Jersey, the kindly notice of my long-time pets, white court plaster and plastic fillings under gold, the aphorism of a perfect gold filling being easier in accomplishment than a good amalgam one, the ——, I like so many things in the three numbers which have come to hand, that were I to mention a tithe of them the brief limits prescribed to me by the Car(r)man of your association would be exceeded, and I shall "draw the line" now at a "misconception," mentioned by the author of "A prize essay on deciduous extraction." The gestation is a healthful one, but his diagnosis of the case is certainly faulty. I conclude this paper with a financial addendum by way of setting a good example to others, and showing a slight appreciation of your excellent labors. Enclosed please find my check in payment of one year's subscription to your journal.

Very truly yours,

J. W. CLOWES, D. D. S.

